# Addressing disparities in the long-term mortality risk in individuals with non-ST segment myocardial infarction (NSTEMI) by diabetes mellitus status: a nationwide cohort study

**DOI:** 10.1007/s00125-024-06281-7

**Published:** 2024-10-03

**Authors:** Andrew Cole, Nicholas Weight, Shivani Misra, Julia Grapsa, Martin K. Rutter, Zbigniew Siudak, Saadiq Moledina, Evangelos Kontopantelis, Kamlesh Khunti, Mamas A. Mamas

**Affiliations:** 1 Keele Cardiovascular Research Group, Centre for Prognosis Research, Institute for Primary Care and Health Sciences, Keele University, Newcastle-under-Lyme, UK; 2 Division of Metabolism, Digestion and Reproduction, Faculty of Medicine, Imperial College London, London, UK; 3 Department of Diabetes, Endocrinology and Metabolism, Imperial College Healthcare NHS Trust, London, UK; 4 Cardiology Department, Guy’s and St Thomas’ NHS Foundation Trust, London, UK; 5 Diabetes, Endocrinology and Metabolism Centre, Manchester University NHS Foundation Trust, National Institute for Health and Care Research (NIHR) Manchester Biomedical Research Centre, Manchester, UK; 6 Division of Diabetes, Endocrinology and Gastroenterology, School of Medical Sciences, Faculty of Biology, Medicine and Health, University of Manchester, Manchester, UK; 7 Institute of Public Health, Jagiellonian University Medical College, Kraków, Poland; 8 Division of Informatics, Imaging and Data Sciences, University of Manchester, Manchester, UK; 9 Diabetes Research Centre, University of Leicester, Leicester, UK; 10 National Institute for Health and Care Research (NIHR) Birmingham Biomedical Research Centre, Birmingham, UK

**Keywords:** Acute myocardial infarction, Cardiovascular epidemiology, Diabetes mellitus, Mortality, Non-ST elevation myocardial infarction, Quality of care

## Abstract

**Aims/hypothesis:**

The aim of this study was to investigate how diabetes mellitus affects longer term outcomes in individuals presenting to hospital with non-ST segment elevation myocardial infarction (NSTEMI).

**Methods:**

We analysed data from 456,376 adults hospitalised between January 2005 and March 2019 with NSTEMI from the UK Myocardial Ischaemia National Audit Project (MINAP) registry, linked with Office for National Statistics death reporting. We compared outcomes and quality of care by diabetes status.

**Results:**

Individuals with diabetes were older (median age 74 vs 73 years), were more often of Asian ethnicity (13% vs 4%) and underwent revascularisation (percutaneous coronary intervention or coronary artery bypass graft surgery) (38% vs 40%) less frequently than those without diabetes. The mortality risk for those with diabetes compared with those without was significantly higher at 30 days (HR 1.19, 95% CI 1.15, 1.23), 1 year (HR 1.28, 95% CI 1.26, 1.31), 5 years (HR 1.36, 95% CI 1.34, 1.38) and 10 years (HR 1.39, 95% CI 1.36, 1.42). In individuals with diabetes, higher quality inpatient care, assessed by opportunity-based quality indicator (OBQI) score category (‘poor’, ‘fair’, ‘good’ or ‘excellent’), was associated with lower mortality rates compared with poor care (good: HR 0.74, 95% CI 0.73, 0.76; excellent: HR 0.69, 95% CI 0.68, 0.71). In addition, compared with poor care, excellent care in the diabetes group was associated with the lowest mortality rates in the diet-treated and insulin-treated subgroups (diet-treated: HR 0.64, 95% CI 0.61, 0.68; insulin-treated: HR 0.69, CI 0.66, 0.72).

**Conclusion/interpretation:**

Individuals with diabetes experience disparities during inpatient care following NSTEMI. They have a higher risk of long-term mortality than those without diabetes, and higher quality inpatient care may lead to better long-term survival.

**Graphical Abstract:**

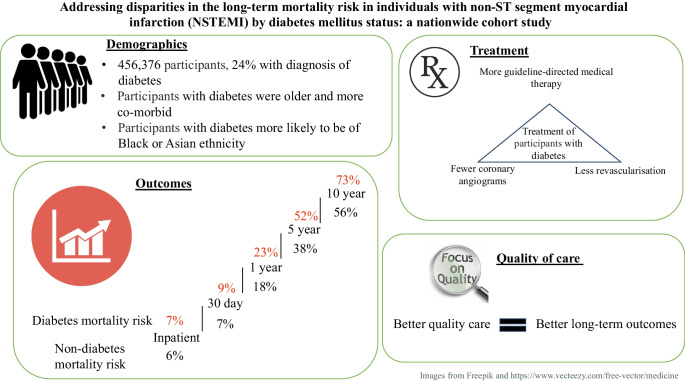

**Supplementary Information:**

The online version of this article (10.1007/s00125-024-06281-7) contains peer-reviewed but unedited supplementary material.


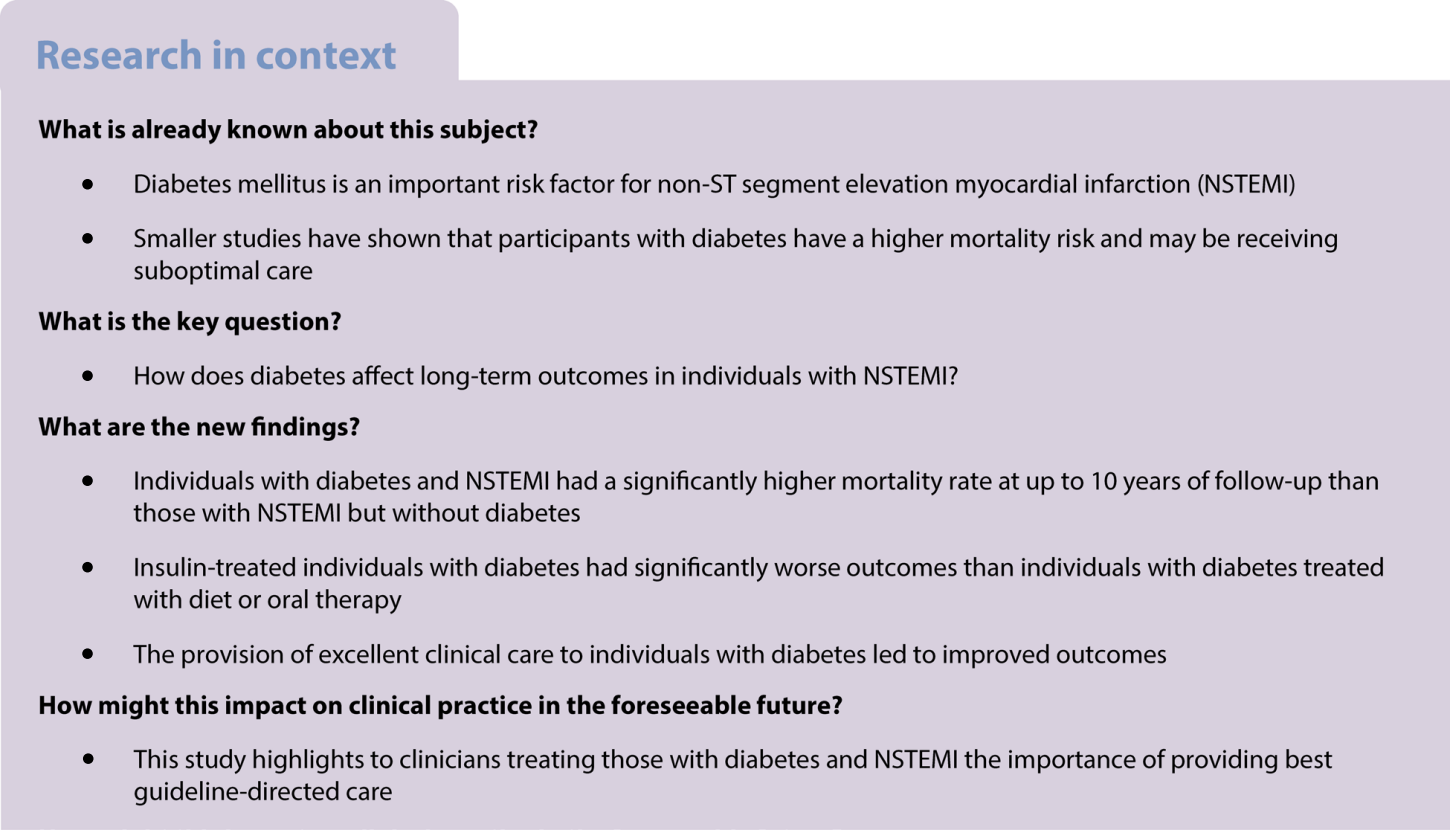


## Introduction

Acute myocardial infarction (AMI) is the leading cause of mortality globally [1], with the incidence of non-ST segment myocardial infarction (NSTEMI), in particular, increasing [2]. Diabetes mellitus is an important risk factor for NSTEMI, with a prevalence within this cohort approaching 25–30% [3]. AMI has a poorer prognosis in individuals with diabetes, with a 50% higher long-term mortality risk than in those without diabetes [4].

Individuals with diabetes are a heterogenous population, with the approaches used for glycaemic management varying from diet and lifestyle modification to oral medications and subcutaneous insulin. The number of interventions for people with diabetes has increased rapidly in the last decade, with improvements in glycaemic monitoring [5], blood glucose control [6] and mortality risk [7], leading to improved cardiovascular outcomes [8].

Historical evidence from smaller cohorts suggests that these recent interventions may have contributed to improved AMI mortality rates [9], but how long-term outcomes have changed within a large contemporary diabetes cohort is unknown. Moreover, there is emerging evidence that people with diabetes presenting with NSTEMI receive suboptimal in-hospital care compared with those with NSTEMI but without diabetes [10].

To address this further, we used data from the Myocardial Ischaemia National Audit Project (MINAP) registry, linked to Office for National Statistics (ONS) mortality data, to compare the in-hospital quality of care that participants receive according to diabetes status, and to assess whether this influences their long-term mortality risk. Our secondary aims were to analyse potential differences in participant outcomes by plasma glucose management type (diet alone, oral therapy or insulin).

## Methods

### Study design

We used the MINAP registry, a prospective national registry of patients admitted to UK hospitals with an acute coronary syndrome (ACS) [11, 12]. The MINAP registry is the UK’s largest AMI registry and consists of 130 variables, including baseline demographics, clinical characteristics, comorbidities, management strategies, pharmacotherapy, in-hospital clinical outcomes and discharge diagnosis [13]. Data are submitted by hospital clinical staff and approximately 90,000 pseudonymised records annually are uploaded to the National Institute for Cardiovascular Outcomes Research (NICOR). Participation in MINAP data entry is mandatory for all hospitals in England and Wales [14]. In-hospital mortality is recorded in the MINAP registry, but for out-of-hospital outcomes we used ONS data, which is the UK’s largest independent provider of official statistics, regularly collecting data on every death registered in the UK, coding deaths according to the ICD-10 (https://icd.who.int/browse10/2019/en) and cause of death from the medical certificate of cause of death.

### Study population

We included individuals admitted with a diagnosis of NSTEMI in any of the 230 participating hospitals in England and Wales between January 2005 and March 2019. The discharge diagnosis of NSTEMI was determined by local clinicians according to presenting history, clinical examination and the results of inpatient investigations in keeping with the consensus document of the Joint European Society of Cardiology (ESC) and American College of Cardiology (ACC) Committee [15]. Participants gender and ethnicity were self-reported during their inpatient admission. Individuals were excluded if they had missing data for our key variables for investigation: diagnosis of diabetes, in-hospital mortality, major adverse cardiovascular events (MACE) and cardiac mortality risk. Participants’ index admission with NSTEMI was used for analysis purposes, with duplicate admissions excluded.

### Subgroup analysis

Subgroup analysis was performed to compare the processes of care and long-term survival rates of participants according to the MINAP categorisation of diabetes management as diet treated, tablet treated or insulin treated.

### Outcomes

#### Primary

The primary outcome was all-cause mortality, specifically 30 day, 1 year, 5 year and 10 year mortality. All-cause mortality was calculated from the date of admission with AMI, as recorded in the MINAP registry, and the date of death, as recorded by the ONS.

#### Secondary

Secondary outcomes of admission for NSTEMI participants were the opportunity-based quality indicator (OBQI) score, which comprises inpatient prescription of aspirin, P2Y_12_ inhibitors, statins, β-blockers, ACE inhibitors or angiotensin receptor blockers (ARBs) and referral to cardiac rehabilitation on discharge [16]. These represent elements of ESC quality metrics and form part of the 2023 ESC AMI guidelines [17]. We classified OBQI scores into four categories: ‘excellent’ (score of **≥**90 and ≤100); ‘good’ (**≥**80 and <90); ‘fair’ (**≥**70 and <80) and ‘poor’ (<70). We also assessed the 2020 ESC Association for Acute Cardiovascular Care (ACVC) quality indicators for NSTEMI [18], including whether participants underwent invasive coronary angiography within 72 h, whether they received dual antiplatelet therapy at discharge, whether they had low molecular weight heparin (LMWH) or fondaparinux prescribed and whether left ventricular function was assessed during admission.

#### Statistical analysis

Demographics, clinical characteristics and crude risks for adverse outcomes by diabetes status were compared using Pearson’s χ^2^ test for categorical variables. Continuous variables were compared using Student’s *t* test if normally distributed and the Wilcoxon rank sum or Kruskal–Wallis test if not. The normality of distribution was assessed using the Shapiro–Wilk test. Continuous variables are presented as medians and IQRs and categorical variables are presented as proportions. Multiple imputations with chained equations (MICE) were used to impute values for variables with missing data. MICE is the best method for dealing with missing data and can provide unbiased estimates even when levels of missing data are significant as well as some protection when the pattern of ‘missingness’ is not random [19]. Kaplan–Meier curves were plotted in Stata 17.0 (StataCorp., USA). Cox regression models were fitted (on ten imputed datasets), adjusted for age, gender, year, ethnicity, hospital, ECG changes, Killip classification, left ventricular function, comorbidities (hypertension, previous stroke, peripheral vascular disease, and asthma or COPD), medication strategy (aspirin, P2Y_12_ inhibitor, ACE inhibitor/ARB, LMWH and warfarin) and invasive coronary angiography (ICA) or revascularisation by percutaneous coronary intervention (PCI) or coronary artery bypass graft (CABG) surgery, to calculate HRs for mortality risks associated with diabetes, with participants without diabetes as the reference. This was performed for mortality over the entire study period and, in addition, sequential Cox regression models with different endpoints were used to model mortality at 30 days, 1 year, 5 years and 10 years. For the analysis of temporal trends in 1 year mortality, study years were organised into 2005–2006, 2007–2008, 2009–2010, 2011–2012, 2013–2014, 2015–2016 and 2017–2018 for ease of presentation.

### Ethics

Secondary use of anonymised MINAP data for research purposes is authorised under NHS research governance arrangements and further supported under Section 251 of NHS Act 2006 (NIGB: ECC1–06(d)/ 2011), which allows researchers to use patient information collected within the dataset for medical research without patient consent. Therefore, formal ethical approval was not sought for this study.

## Results

**Fig. 1:**
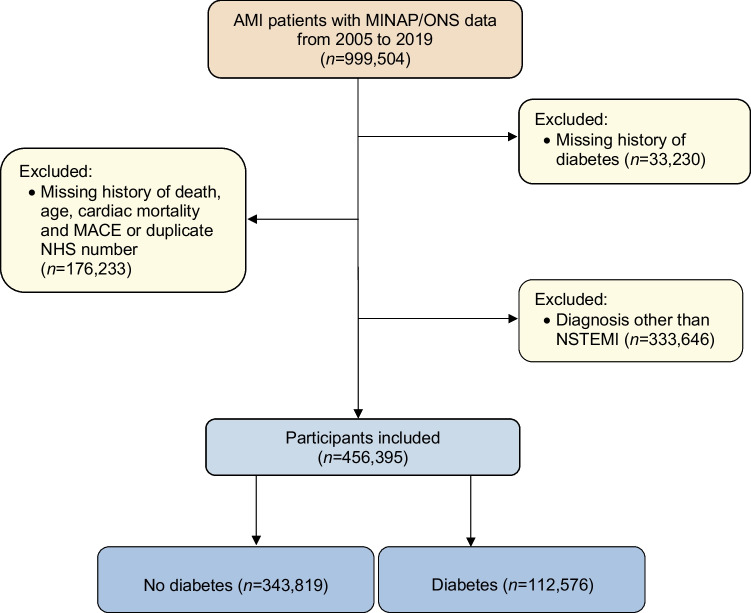
Flow diagram of participant inclusion

After applying relevant exclusion criteria, 456,395 individuals admitted to hospital in England and Wales between January 2005 and March 2019 with a diagnosis of NSTEMI were included in the analysis (Fig. 1). In total, 112,576 (25%) had a diagnosis of diabetes. Of those with diabetes, 19,597 (17%) were managed with diet only, 58,828 (52%) were managed with oral hypoglycaemic medication and 34,130 (30%) were treated with insulin. A total of 21 participants did not have a treatment modality recorded. The mean (SD) duration of follow-up for included participants was 1976 (1560) days.

### Demographic comparison between NSTEMI participants with and without diabetes

Compared with participants without diabetes, those with diabetes were older (median age 74 [IQR 64.7–81.2] vs 73 [IQR 61.2–82.6]) years) and were more likely to be of Asian (13% vs 4%, *p*<0.001) or Black (2% vs 1%, *p*<0.001) ethnicity (Table 1). Participants with diabetes were also more likely to present with common cardiovascular risk factors, including hypercholesterolaemia (46% vs 31%, *p*<0.001), chronic kidney disease (15% vs 6%, *p*<0.001), hypertension (69% vs 50%, *p*<0.001) and a history of previous AMI (36% vs 24%, *p*<0.001). Furthermore, participants with diabetes were more likely to have had previous PCI (16% vs 10%, *p*<0.001) and CABG (13% vs 6%, *p*<0.001). During admission, those with diabetes were more likely to develop severe left ventricular systolic dysfunction (LVSD) (defined as an ejection fraction <30%; 10% vs 6%, *p*<0.001) and present with a higher Killip class (basal crepitations 19% vs 14%,* p*<0.001; pulmonary oedema 9% vs. 5%, *p*<0.001). On admission, participants with diabetes were less likely to be admitted to a cardiology ward (50% vs 51%, *p*<0.001).

**Table 1:** Demographic comparison between NSTEMI participants with and without diabetes

Variable	No diabetes (*N*=343,819)	Diabetes (*N*=112,576)	*p* value
Age (years)	73.0 (61.2–82.6)	74.0 (64.7–81.2)	<0.001
Female	126,439/343,819 (37)	40,524/112,576 (36)	<0.001
BMI (kg/m^2^)	26.6 (23.6–30.1)	28.7 (25.2–32.8)	<0.001
Ethnicity			
White	167,713/176,810 (95)	53,392/63,019 (85)	<0.001
Asian	7254/176,810 (4)	8287/63,019 (13)	<0.001
Black	1496/176,810 (1)	1157/63,019 (2)	<0.001
Mixed	347/176,810 (0)	183/63,019 (0)	<0.001
Killip class			
Basal crepitations	22,375/160,119 (14)	11,145/58,341 (19)	<0.001
Pulmonary oedema	7352/160,119 (5)	5267/58,341 (9)	<0.001
Cardiogenic shock	818/160,119 (1)	375/58,341 (1)	<0.001
GRACE score			
High risk (>140)	94,417/155,925 (61)	39,642/56,752 (70)	<0.001
Intermediate risk (109–140)	41,022/155,925 (26)	12,541/56,752 (22)	<0.001
Low risk (<109)	20,486/155,925 (13)	4569/56,752 (8)	<0.001
ECG ST changes	257,557/330,261 (78)	85,082/108,232 (79)	<0.001
Previous smoker	118,190/326,146 (36)	43,221/105,434 (41)	<0.001
Current smoker	76,966/326,146 (24)	17,628/105,434 (17)	<0.001
CCF	21,175/321,638 (7)	12,578/105,073 (12)	<0.001
Hypercholesterolaemia	98,390/320,898 (31)	47,881/104,876 (46)	<0.001
Cerebrovascular disease	29,154/322,263 (9)	14,011/105,233 (13)	<0.001
CKD^a^	19,010/321,714 (6)	15,488/105,069 (15)	<0.001
History of angina	87,721/324,122 (27)	42,685/105,771 (40)	<0.001
Peripheral vascular disease	13,094/319,051 (4)	9351/104,319 (9)	<0.001
Hypertension	162,117/326,762 (50)	74,192/107,030 (69)	<0.001
Asthma/COPD	54,685/320,128 (17)	19,936/104,804 (19)	<0.001
Previous AMI	77,482/327,216 (24)	39,587/106,685 (37)	<0.001
Previous PCI	31,905/322,547 (10)	17,626/105,048 (17)	<0.001
Previous CABG	20,811/323,090 (6)	13,732/105,489 (13)	<0.001
Family history of CAD	78,311/263,329 (30)	21,578/83,434 (26)	<0.001
Heart rate (bpm)	78 (66–92)	82 (70–97)	<0.001
Systolic BP (mmHg)	140 (121–159)	140 (122–159)	<0.001
LV function^b^			
Good	92,376/242,937 (38)	27,731/81,573 (34)	<0.001
Moderate	39,772/242,937 (16)	16,759/81,573 (21)	<0.001
Severe	15,406/242,937 (6)	7772/81,573 (10)	<0.001
Cardiac arrest	11,441/334,765 (3)	4237/110,071 (4)	<0.001
Admission under cardiologist	149,848/335,759 (45)	48,780/109,796 (44)	0.075
Admission to cardiology ward^c^	173,509/341,790 (51)	55,665/111,884 (50)	<0.001

Continuous variables are expressed as median (IQR) and categorical variables as proportions (%). Denominators represent the total number of participants with a data point collected; numerators represent the number of those participants for whom the variable of interest was present

^a^CKD is recorded in the MINAP registry as a serum creatinine level chronically elevated above 200 µmol/l

^b^Good left ventricular function was defined as an ejection fraction (EF) ≥50%, moderate LV function as an EF 30–49% and severe LV function as an EF <30%

^c^‘Admission to cardiology ward’ is a composite of admission to a coronary care unit or a general cardiology ward

bpm, beats per minute; CAD, coronary artery disease; CCF, congestive cardiac failure; CKD, chronic kidney disease, GRACE, Global Registry of Acute Coronary Events; LV, left ventricular

### Management strategies and unadjusted clinical outcomes for NSTEMI participants with and without diabetes

Participants with diabetes were less likely than those without diabetes to undergo ICA (59% vs 63%, *p*<0.001) and subsequent revascularisation by PCI or CABG (38% vs 40%, *p*<0.001) (Table 2). Those with diabetes were also more likely than those without diabetes to receive statins (89% vs 82%, *p*<0.001), ACE inhibitors/ARBs (78% vs 72%, *p*<0.001) and β-blockers (74% vs 71%, *p*<0.001). Unadjusted in-hospital MACE (8% vs 7%, *p*<0.001), in-hospital mortality (7% vs 6%, *p*<0.001) and a circulatory cause of death (53% vs 48%, *p*<0.001) were more prevalent in the diabetes group than the non-diabetes group.

**Table 2:** Management strategy and clinical outcome comparison between NSTEMI participants with and without diabetes

Variable	No diabetes (*N*=343,819)	Diabetes (*N*=112,576)	*p* value
LMWH	174,507/294,391 (59)	54,938/95,342 (58)	<0.001
Fondaparinux	105,916/254,943 (42)	35,092/84,963 (41)	0.168
Warfarin	17,215/289,822 (6)	7181/94,282 (8)	<0.001
Unfractionated heparin	39,199/288,584 (14)	13,127/93,883 (14)	<0.001
Glycoprotein 2b/3a inhibitor	12,078/294,815 (4)	3231/95,687 (3)	<0.001
Intravenous nitrate	39,181/289,761 (14)	14,912/94,278 (16)	<0.001
Furosemide	76,681/290,886 (26)	40,297/94,928 (42)	<0.001
MRAs	12,897/214,672 (6)	7128/73,281 (10)	<0.001
Aspirin	251,540/262,190 (96)	80,774/84,543 (96)	<0.001
P2Y_12_ inhibitors	287,017/333,534 (86)	94,678/109,735 (86)	0.035
Statins	277,408/337,074 (82)	98,352/110,561 (89)	<0.001
ACE inhibitors/ARBs	242,482/335,576 (72)	86,217/110,032 (78)	<0.001
β-blockers	238,978/334,373 (71)	81,327/109,868 (74)	<0.001
ICA	207,341/333,173 (62)	63,074/108,814 (58)	<0.001
PCI	101,435/273,396 (37)	29,215/88,017 (33)	<0.001
CABG surgery	9178/273,396 (3)	4054/88,017 (5)	<0.001
Revascularisation (CABG surgery/PCI)	110,613/273,396 (40)	33,269/88,017 (38)	<0.001
In-hospital mortality	20,176/343,819 (6)	7799/112,576 (7)	<0.001
30 day mortality	24,042/343,819 (7)	9507/112,576 (8)	<0.001
1 year mortality	60,580/343,819 (18)	26,427/112,576 (23)	<0.001
5 year mortality	104,666/276,183 (38)	44,407/85,854 (52)	<0.001
10 year mortality	84,320/151,120 (56)	31,801/43,243 (74)	<0.001
Inpatient cardiac mortality	15,370/343,819 (4)	6079/112,576 (5)	<0.001
Reinfarction	3146/310,448 (1)	1276/102,401 (1)	<0.001
Major bleeding	5020/332,625 (2)	1917/109,583 (2)	<0.001
MACE^a^	22,597/343,819 (7)	8750/112,576 (8)	<0.001
Circulatory cause of death	80,553/167,870 (48)	35,824/68,055 (53)	<0.001

Data are presented as proportions (%). Denominators represent the total number of participants with a data point collected; numerators represent the number of those participants for whom the variable of interest was present

^a^MACE was defined as a composite endpoint of in-hospital death and reinfarction

MRA, mineralocorticoid receptor antagonist

### Quality indicators for NSTEMI participants with and without diabetes

Participants with diabetes were less likely than those without diabetes to receive ICA within 72 h (57% vs 66%, *p*<0.001) (Table 3). In cases of moderate to severe LVSD, those with diabetes were more likely to be started on ACE inhibitors/ARBs (81% vs 77%, *p*<0.001) and β-blockers (77% vs 74%, *p*<0.001) than those without diabetes. The mean OBQI score for participants with diabetes was higher than that in those without diabetes (81.5 vs 79.2) *p*<0.001).

**Table 3:** Quality indicators (ESC ACVC and OBQI) for NSTEMI participants with and without diabetes

Variable	No diabetes (*N*=343,819)	Diabetes (*N*=112,576)	*p* value
ICA received within 72 h	29,143/44,116 (66)	9199/16,133 (57)	<0.001
LV function recorded in notes	147,554/242,937 (61)	52,262/81,573 (64)	<0.001
Fondaparinux or LMWH received	252,729/289,433 (87)	79,968/93,898 (85)	<0.001
DAPT received on discharge	219,065/272,812 (80)	70,728/88,443 (80)	0.059
ACEi or ARB received on discharge for those with moderate to severe LVSD	41,737/54,813 (76)	19,068/23,696 (80)	<0.001
β-blocker received on discharge for those with moderate to severe LVSD	38,714/52,649 (74)	18,032/23,356 (77)	<0.001
Composite all/none score^a^	204,359/267,342 (76)	66,138/86,413 (77)	<0.001
Composite all/none score for those with moderate to severe LVSD^b^	33,932/43,107 (79)	15,009/18,925 (79)	<0.001
OBQI			
Mean OBQI score	79.2	81.5	<0.001
Cardiac rehabilitation	244,941/312,079 (78)	77,004/101,596 (76)	<0.001

Data are expressed as proportions (%) unless indicated otherwise. Denominators represent the total number of participants with a data point collected; numerators represent the number of those participants for whom the variable of interest was present

^a^Composite score of receipt of low-dose aspirin, a P2Y_12_ inhibitor and a statin

^b^Opportunity-based care score. The score consisted of six evidence-based processes of care: prescription of aspirin, a thienopyridine inhibitor, a β-blocker, an ACE inhibitor and a hydroxymethylglutaryl-coenxyme A (HMG CoA) reductase enzyme inhibitor (statin) and enrolment onto a cardiac rehabilitation programme at the time of discharge. The score reflects the number of care opportunities fulfilled at each hospital (numerator) divided by the number of opportunities to provide care (denominator). Interventions that were contraindicated, not applicable or not indicated in or declined by individual participants were excluded from both the numerator and the denominator

ACEi, ACE inhibitor; EF, ejection fraction

Participants with diabetes treated with insulin were younger (median [IQR] age 72.1 [63.2–79.3] years) than tablet-treated participants (74.1 [64.6–81.1] years) and diet-treated participants (77.0 [67.5–84.1] years) (ESM Table 1). Tablet-treated participants most often underwent ICA (tablet-treated: 62%; diet-treated: 54%; insulin-treated: 57%). Tablet-treated participants were also most likely to undergo revascularisation by PCI/CABG surgery (tablet-treated: 40%, diet-treated: 34%; insulin-treated: 36%) (ESM Table 2). Insulin-treated participants were least likely to receive an angiogram within 72 h (insulin-treated: 53%; diet-treated: 58%; tablet-treated: 59%). Quality of care according to the mean OBQI score was highest in the tablet-treated group (82.4 vs insulin-treated 81.4 and diet-treated 79.2) (ESM Table 3).

### Long-term mortality analysis

**Fig. 2:**
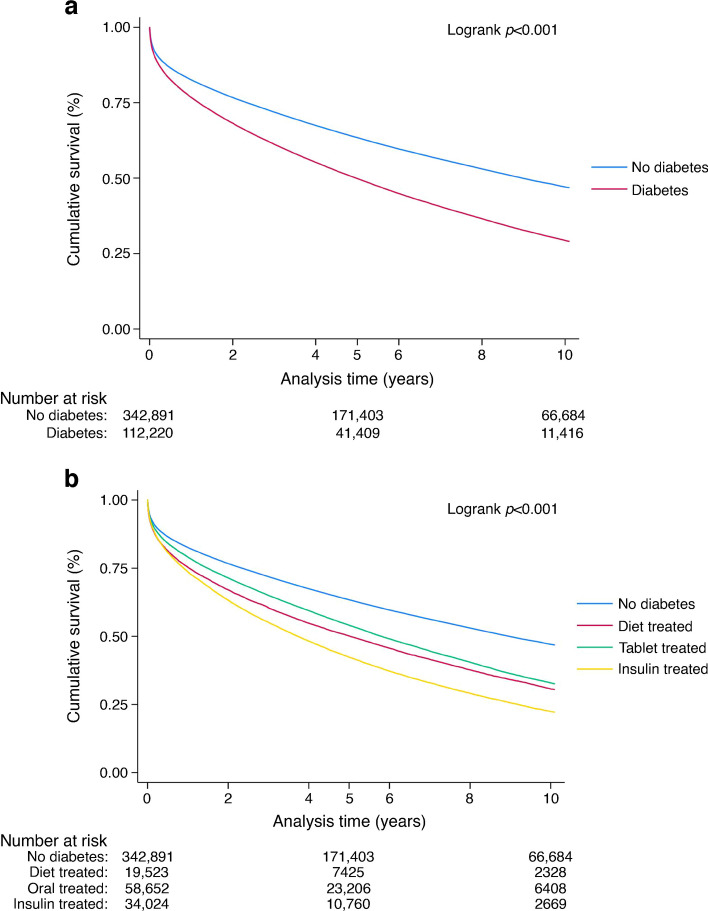
Kaplan–Meier survival curves for participants with diabetes compared with participants without diabetes. (**a**) All participants with diabetes compared with those without diabetes; (**b**) diabetes subgroups based on treatment modality compared with those without diabetes. The MINAP registry does not record whether individuals have type 1 or type 2 diabetes, only whether they are managed with diet, medication or insulin. Individuals with insulin-dependent diabetes will therefore represent a combination of type 1 and type 2 diabetes. The number at risk at time point 0 is slightly lower than the total study number as it does not include those who died on day 0 of admission

Participants with diabetes had a higher unadjusted mortality rate than those without diabetes at all time points: 30 days, 9% vs 7% (*p*<0.001); 1 year, 23% vs 18% (*p*<0.001); 5 years, 52% vs 38% (*p*<0.001); and 10 years, 73% vs 56% (*p*<0.001), respectively (Table 2). Similarly, in the multivariate model, mortality risk remained higher for those with diabetes than those without diabetes at 30 days (HR 1.19, 95% CI 1.15, 1.23, *p*<0.001), 1 year (HR 1.28, 95% CI 1.26, 1.31, *p*<0.001), 5 years (HR 1.36, 95% CI 1.34, 1.38, *p*<0.001) and 10 years (HR 1.39, 95% CI 1.36, 1.42, *p*<0.001) (Table 4, Fig. 2).

**Table 4:** Survival analysis for participants with diabetes compared with participants without diabetes

Outcome variable	Adjusted HR (95% CI)	*p* value
30 day mortality	1.19 (1.15, 1.23)	<0.001
1 year mortality	1.28 (1.26, 1.31)	<0.001
5 year mortality	1.36 (1.34, 1.38)	<0.001
10 year mortality	1.39 (1.36, 1.42)	<0.001

HRs were adjusted for age, gender, ethnicity, year, heart rate, BP, hospital, comorbid conditions (hypertension, hypercholesterolaemia, history of asthma or COPD, history of cerebral vascular accident or peripheral vascular disease), pharmacotherapy (prescription of LMWH, warfarin, aspirin and P2Y_12_ inhibitor and ACE inhibitor/ARB), cardiac arrest and procedures including coronary angiography during admission and revascularisation (by PCI or CABG surgery during admission)

**Fig. 3:**
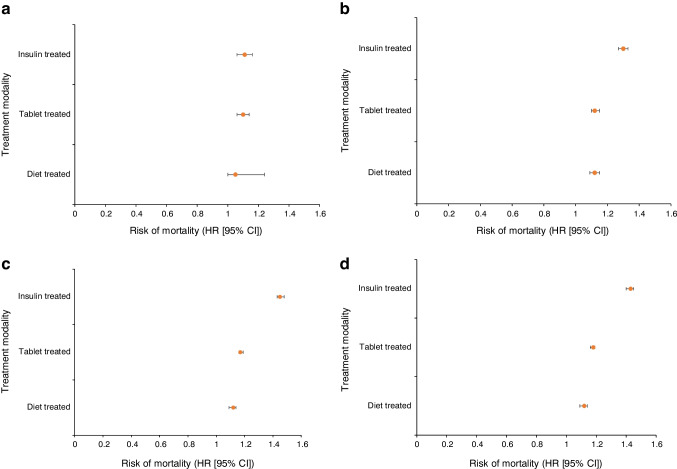
Forest plots of mortality for the subgroups of participants with diabetes compared with participants without diabetes. (**a**) 30 day mortality; (**b**) 1 year mortality; (**c**) 5 year mortality; (**d**) 10 year mortality. Adjusted Cox regression model adjusted for age, gender, year, ethnicity, hospital, ECG changes, Killip classification, comorbidities (previous stroke, peripheral vascular disease, and asthma or COPD), medication strategy (aspirin, P2Y_12_ inhibitor, ACE inhibitor/ARB, LMWH and warfarin) and ICA or revascularisation by PCI or CABG surgery

Subgroup analysis showed that insulin-treated participants had the highest mortality rate at each time point (Table 5, Fig. 3). A landmark analysis of these data (excluding mortality within 30 days of admission) revealed similar findings (ESM Fig. 1).

**Table 5:** Survival analysis for subgroups of participants with diabetes based on treatment modality compared with participants without diabetes

Outcome variable	Adjusted HR (95% CI)					
	Diet-treated diabetes (*N*=18,689)	*p* value	Tablet-treated diabetes (*N*=57,826)	*p* value	Insulin-treated diabetes (*N*=33,728)	*p* value
30 day mortality	1.10 (1.04, 1.18)	0.003	1.18 (1.13, 1.24)	<0.001	1.27 (1.20, 1.34)	<0.001
1 year mortality	1.17 (1.13, 1.22)	<0.001	1.21 (1.18, 1.25)	<0.001	1.49 (1.45, 1.54)	<0.001
5 year mortality	1.16 (1.13, 1.20)	<0.001	1.26 (1.24, 1.29)	<0.001	1.72 (1.68, 1.76)	<0.001
10 year mortality	1.19 (1.15, 1.24)	<0.001	1.30 (1.27, 1.33)	<0.001	1.77 (1.72, 1.82)	<0.001

HRs were adjusted for age, gender, ethnicity, year, hospital, heart rate, BP, hospital, comorbid conditions (hypertension, hypercholesterolaemia, history of asthma or COPD, history of cerebrovascular accident or peripheral vascular disease), pharmacotherapy (prescription of LMWH, warfarin, aspirin and P2Y_12_ inhibitor and ACE inhibitor/ARBs) and procedures including coronary angiography during admission and revascularisation (by PCI or CABG during admission)

The MINAP registry does not record whether patients have type 1 or type 2 diabetes, only whether they are managed with diet, medication or insulin. Individuals with insulin-dependent diabetes will therefore represent a combination of type 1 and type 2 diabetes

**Fig. 4:**
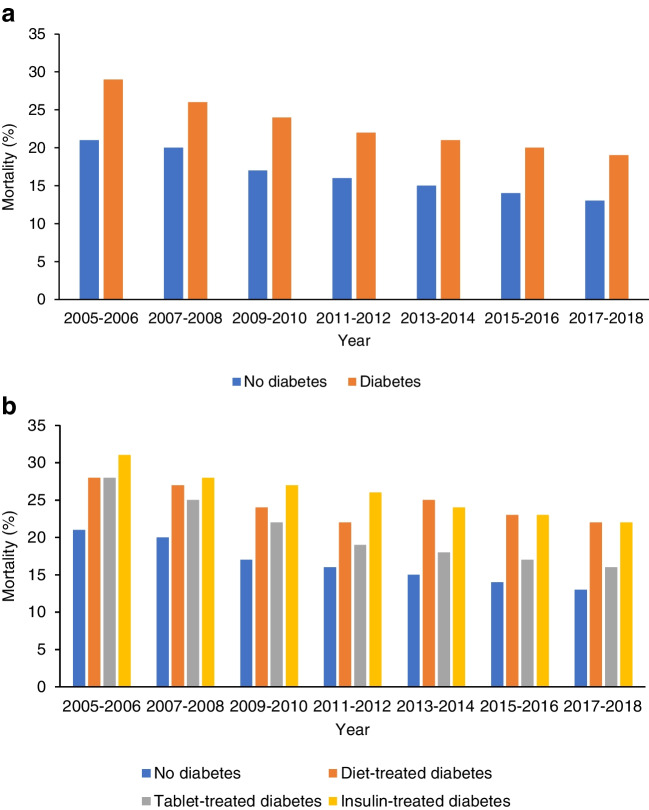
Temporal trends in 1 year mortality. (**a**) All participants with diabetes compared with those without diabetes; (**b**) diabetes subgroups based on treatment modality compared with those without diabetes

Temporal trends in absolute risk for 1 year mortality improved similarly for participants with and without diabetes (2005–2006: 29% vs 21%; 2017–2018: 19% vs 13%, respectively; Fig. 4a). This temporal change in 1 year mortality risk was greatest in the tablet-treated subgroup and lowest in the diet-treated subgroup (2005–2006: diet-treated 28%, tablet-treated 28%, insulin-treated 31%; 2017–2018: diet-treated 22%, tablet-treated 16%, insulin-treated 22%; Fig. 4b).

### Subgroup quality of care analysis

**Fig. 5:**
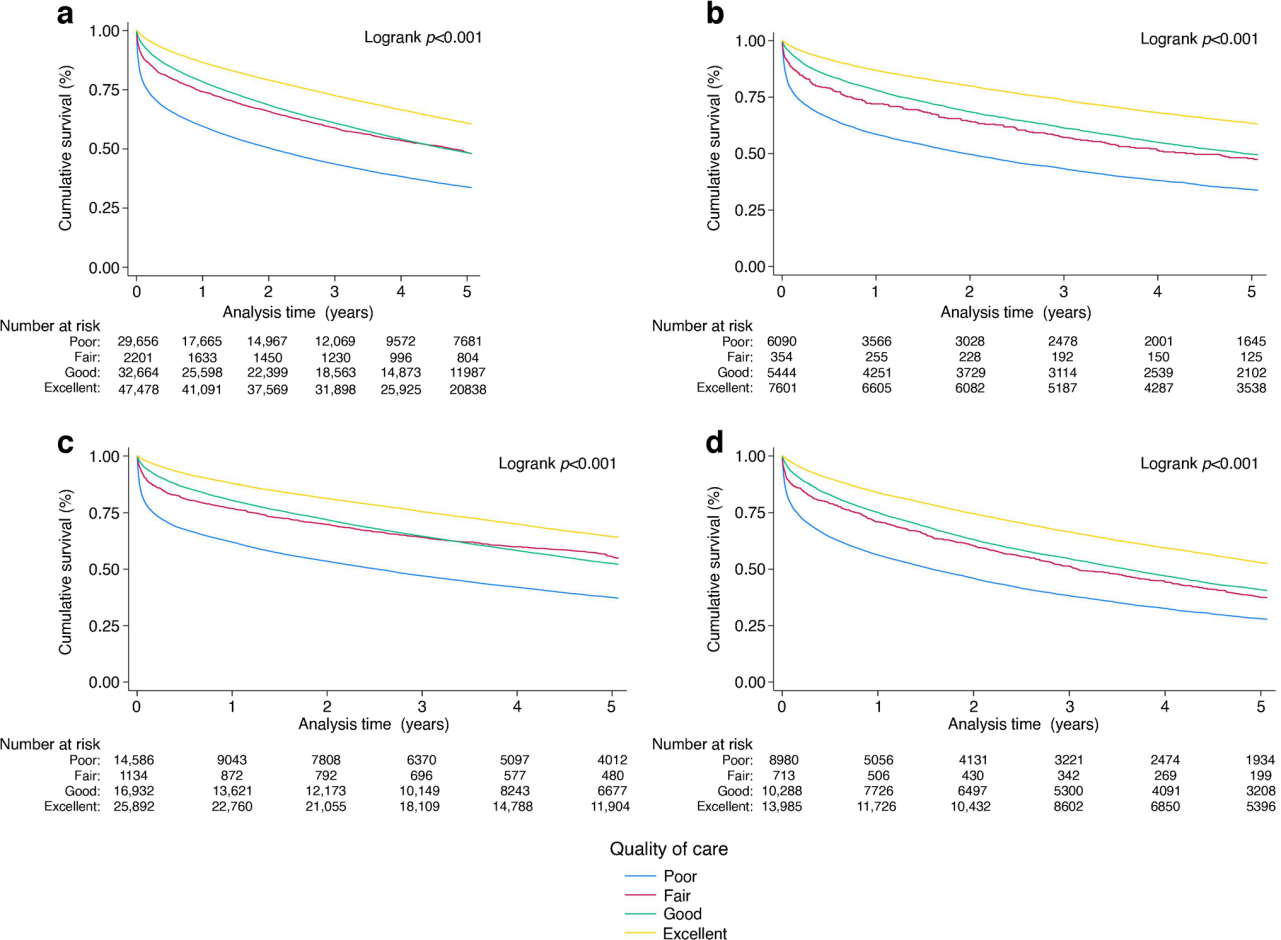
Kaplan–Meier survival curves showing the impact of inpatient quality of care according to OBQI score on survival rates in participants with diabetes. (**a**) All participants with diabetes; (**b**) diet-treated participants with diabetes; (**c**) tablet-treated participants with diabetes; (**d**) insulin-treated participants with diabetes. The number at risk at time point 0 is slightly lower than the total study number as it does not include those who died on day 0 of admission. Comparison with ‘poor care’ according to OBQI score: (**a**) fair: HR 1.06 (95% CI 1.02, 1.16), *p*=0.007; good: HR 0.74 (95% CI 0.73, 0.76), *p*<0.001; excellent: HR 0.69 (95% CI 0.68, 0.71), *p*<0.001; (**b**) fair: HR 1.05 (95% CI 0.95, 1.16), *p*=0.293; good: HR 0.71 (95% CI 0.67, 0.76), *p*<0.001; excellent: HR 0.64 (95% CI 0.61, 0.68), *p*<0.001; (**c**) fair: HR 1.09 (95% CI 1.03, 1.15), *p*=0.004; good: HR 0.75 (95% CI 0.72, 0.77), *p*<0.001; excellent: HR 0.71 (95% CI 0.68, 0.74), *p*<0.001; (**d**) fair: HR 1.03 (95% CI 0.96, 1.10), *p*=0.434; good: 0.75 (95% CI 0.72, 0.78), *p*<0.001; excellent: HR 0.69 (95% CI 0.66, 0.72), *p*<0.001

Among all participants with diabetes, higher quality of inpatient care according to OBQI score was associated with lower multivariable-adjusted risks of long-term mortality. Good care (HR 0.74, 95% CI 0.73, 0.76, *p*<0.001) and excellent care (HR 0.69, 95% CI 0.68, 0.71, *p*<0.001) were associated with better survival rates than poor care (Fig. 5a). Compared with poor care, excellent care was associated with the greatest survival advantage in diet-treated (HR 0.64, 95% CI 0.61, 0.68, *p*<0.001) and insulin-treated participants (HR 0.69, 95% CI 0.66, 0.72, *p*<0.001) (Fig. 5b–d).

## Discussion

Our comprehensive analysis of over 450,000 individuals admitted with NSTEMI, with follow-up of up to 10 years, reveals important disparities in care and long-term outcomes between those with diabetes and those without diabetes. First, those with diabetes were less likely to undergo invasive investigation with ICA or revascularisation but were more likely to receive guideline-directed medical therapy including statins, β-blockers and ACE inhibitors. Reassuringly, the quality of care received overall as measured by the OBQI score was better for those with diabetes than for those without diabetes. Second, the risk of mortality at 30 days, 1 year and up to 10 years of follow-up was significantly higher for the diabetes population, but, importantly, receipt of higher quality guideline-recommended care was associated with better long-term mortality risk across all groups of individuals with diabetes.

Previous studies have reported disparities in AMI care for people with diabetes [16, 20]; however, these studies mostly investigated ACS, broadly including both individuals with STEMI and those with NSTEMI [21]. Management of individuals with STEMI is more protocolised and typically managed within specialist centres, whereas the management of NSTEMI shows greater heterogeneity [22]. It is therefore difficult to generalise ACS results to NSTEMI cohorts. Additionally, many previous analyses were limited by including smaller cohorts and shorter periods of follow-up and did not consider diabetes groups based on their treatment regimen. Our study included a large cohort of individuals with diabetes, with up to 10 years’ follow-up for a proportion of the study participants, which far exceeds that of comparable studies.

We observed that participants with diabetes were more likely to present acutely unwell with NSTEMI, with a higher Killip class and a higher proportion of moderate to severe LVSD. Similar, to Killip class, left ventricular dysfunction is an independent risk factor for worse outcomes following PCI [23]. Those with diabetes were also more likely to have prevalent comorbidities such as chronic kidney disease (CKD). CKD increases bleeding risk from invasive therapy [24] and may require caution from operators providing invasive procedures in which nephrotoxic contrast media is used [25]. Furthermore, individuals with CKD and diabetes are more likely to present with diffuse multivessel coronary artery disease that is not amenable to PCI [26]. These factors may explain why people with diabetes do not receive timely revascularisation, but it must be highlighted that this is in direct contrast to the 2018 ESC/European Association for Cardio-Thoracic Surgery (EACTS) guidelines on ACS revascularisation, which recommend early revascularisation in people with diabetes [27]. The clinical implications of this delay remain controversial, with some studies suggesting that an early invasive strategy leads to improved long-term mortality risks in people with NSTEMI [28]. However, other contemporary trials suggest that there is no mortality benefit of early intervention in those with NSTEMI, although these studies reported lower rates of angina and shorter hospitalisations [29]. Of interest, despite our diabetes cohort presenting as more unwell and with additional comorbidities, the overall rate of cardiogenic shock within the whole cohort was low at 1%, this is lower than the estimated rates of cardiogenic shock post NSTEMI of 2.5% [30]. This difference may be explained by advancements in therapies or a lack of identification or clear documentation from treating clinicians.

Canto et al showed in a cohort of 434,877 individuals with myocardial infarction that diabetes was more common in those presenting with atypical chest pain (32.6% vs 25.4%) [31]. This may also directly contribute to invasive therapy delays. Our observed lower rate of invasive management could also represent a known selection bias that occurs whereby lower risk individuals are more likely to receive guideline-directed invasive therapy than individuals at higher risk over concerns of complications during procedures, a so-called risk–treatment paradox [32]. In contrast to this, individuals with diabetes in this study were more likely to be prescribed guideline-directed medical therapy such as β-blockers, ACE inhibitors and statins.

Considering the effect of quality of care further, participants with diabetes were less likely to be admitted to a specialist cardiology ward. Being cared for on a specialist cardiology ward leads to increased delivery of guideline-directed medical therapy [33]. Individuals are also more likely to receive specialist investigations such as echocardiograms and cardiac MRI, which allow rapid diagnosis and treatment of post-infarct complications and prompt more aggressive titration of heart failure therapy [34]. Additionally, specialists are more likely to understand and use local referral pathways on discharge for cardiac rehabilitation. Previous studies have shown the importance of cardiac rehabilitation in post-AMI care [35] and that non-cardiac specialists are less likely to refer eligible patients for these services [36].

Outcomes for people with diabetes have consistently been shown to be worse post AMI than for those without diabetes. Donahoe et al showed in a multinational cohort of 62,036 individuals with ACS from 1997 to 2006 that the mortality risk was significantly higher at 30 days and 1 year of follow-up in those with diabetes than in those without diabetes [37]. Similarly, Fojt et al showed in a Polish-based cohort of 58,394 individuals with AMI from 2009 to 2012 that the presence of diabetes was associated with a higher risk of mortality up to 3 years of follow-up [38]. Our study builds on this previous evidence in key areas, with up to 10 years of follow-up for mortality events. We demonstrate that in UK individuals with NSTEMI, those with diabetes have a higher risk of mortality, which is 16% greater at 5 years and 11% greater at 10 years of follow-up than in individuals without diabetes. It is interesting that, over this long follow-up duration, the disparity in mortality between those with diabetes and those without remains, suggesting that the impact of diabetes on NSTEMI outcomes persists long after the index cardiac event.

Furthermore, our study is also the first to compare quality of NSTEMI care and show how this is related to long-term mortality risks. Many factors that contribute to higher mortality risk in people with diabetes, such as common cardiovascular risk factors, exist at the point of index NSTEMI presentation and are non-modifiable by the hospital specialist caring for them. However, the quality of care that clinicians deliver is a modifiable aspect of care and this study demonstrates the positive association between higher quality inpatient care and long-term mortality risks in UK NSTEMI patients. Our study highlights the importance of clinicians delivering guideline-led, high-quality inpatient care to all those with diabetes.

Our study was able to stratify a large cohort of individuals by diabetes treatment modality and follow these individuals over a long time period. We show that, over time, mortality trends in our population with diabetes improved and that individuals managed with insulin had significantly higher mortality over all time periods. This may have occurred for several reasons. First, medications used by those with diabetes, such as metformin, glucagon-like peptide-1 (GLP-1) receptor agonists and sodium–glucose cotransporter 2 (SGLT2) inhibitors, all have strong cardioprotective qualities [39–41], although the MINAP registry does not yet collect data on these medications. It is also possible that once people are diagnosed with diabetes they begin to address other comorbid factors such as body weight, diet, exercise and smoking. It is also possible that improved recognition and prompt medical management of diabetes increases the amount of contact that these individuals have with medical professionals, increasing the opportunity for preventative intervention. When considering why insulin-treated individuals have the poorest outcomes, it is important to highlight that our insulin-treated cohort was made of two distinct groups, those with type 1 diabetes and those with type 2 diabetes of longer duration who have failed to establish optimal glycaemic management with other therapies. This group will have the highest rate of intrinsic insulin resistance. Studies have shown that insulin resistance advances the atherosclerotic process [42], with individuals with diabetes being more likely to present with multivessel involvement [43] and having a propensity for in-stent restenosis [44]. This in turn could partly explain their higher short- and long-term mortality risk [45] and our finding that individuals with diabetes more often had a history of previous AMI.

To our knowledge, this is the first study to investigate the relationship between inpatient quality of care and long-term mortality risks in individuals with diabetes and NSTEMI in a large, national healthcare setting. We suggest that further studies should focus on identifying and addressing the disparities in access to guideline-directed medical therapy, cardiac rehabilitation referral and prompt invasive investigations and therapies in the highest risk individuals with diabetes post AMI to enable major improvements to be made in the long-term survival rates of this growing population.

### Strengths

There are several other major strengths to this study. The MINAP registry collects robust data, with many variables recorded from all those presenting to hospital with NSTEMI in the UK. This allows for regional differences within the UK to be balanced out and thus our results are more likely to be representative of other publicly funded healthcare models globally. Our post-discharge mortality data from the ONS, with a long mean duration of follow-up of 1900 days, enabled us to assess mortality risks. Furthermore, our study has the additional benefit of studying patients within the UK where healthcare is free at the point of use. Compared with private healthcare systems, where up to a quarter of patients avoid hospital because of the high associated costs, our study is likely to have captured a more complete spectrum of diabetes/NSTEMI presentations [46]. Overall, this study reports on a large, contemporary cohort with a far greater follow-up duration than in previous studies in this patient group.

### Limitations

There are also several important limitations to consider in this study. The MINAP database is limited in similar ways to other national databases. First, there is no external validation of data inputs. Second, although the MINAP database collects many variables of interest, it does not collect data on frailty, rationale for treatment options or an exhaustive list of comorbidities. Additionally, the MINAP database has strict definitions for recorded comorbidities. For example, CKD was defined in this study as a creatinine level of >200 µmol/l. Therefore, CKD defined by MINAP encompasses those with moderate CKD to those established on dialysis. This limited our ability to subclassify participants depending on their degree of kidney disease. Similarly, MINAP defines hypercholesterolaemia as an ‘elevation of serum cholesterol requiring dietary or drug treatment’ and does not provide guidance on severity. Additionally, although the MINAP dataset should only include type 1 myocardial infarctions, there will undoubtedly be some misclassification with some type 2 myocardial infarctions also included. This distinction affects the use of invasive therapies as well as clinical outcomes.

It is important to recognise that diabetes recording in the MINAP registry occurs at the point of NSTEMI diagnosis. This database does not record when individuals were diagnosed with diabetes and therefore our cohort will contain those who were diagnosed prior to admission and those who, when admitted, had an index diagnostic HbA_1c_ level recorded. These newly diagnosed individuals are often recorded as ‘diet-controlled’; however, they will have had no prior diabetes intervention or advice. This group will be heterogeneous and this may explain the difference in mortality between the diet- and tablet-treated groups. Equally, this database collects generic information around insulin use in individuals with diabetes. It does not capture whether insulin-treated individuals have type 1 or type 2 diabetes. There is likely to be a subgroup of individuals with type 2 diabetes in our insulin-treated group who are also taking a range of oral agents. We are therefore unable to assess if a combination of methods to control plasma glucose or the specific aetiology of diabetes makes a difference to outcomes in NSTEMI. With regard to our assessment of mortality risk, we have no data on longer term participant adherence to medications, nor were we able to delineate sub-cohorts of individuals who transition through different diabetes treatments over time. Additionally, there was a possible selection bias in our quality-of-care groups because, to receive a referral to cardiac rehabilitation, patients need to be fit enough to attend and have sufficient renal function for ACE inhibitor initiation, and their prognosis needs to be good enough to start on statins.

Although we present prospective data and our modelling adjusted for many important confounding variables, these observational data have the potential for residual confounding and therefore caution is needed when making causal inferences. We stratified our participants by treatment modality but were not able to identify individuals treated with SGLT2i or GLP-1 receptor agonists. Future research should aim to assess the potential benefits of these agents in the setting of NSTEMI.

### Conclusion

Our nationwide analysis of the long-term outcomes of over 450,000 UK individuals presenting with NSTEMI shows that there are stark disparities in NSTEMI management between those with and those without diabetes. Those with diabetes had an elevated risk of mortality at all study time points up to 10 years compared with those without diabetes. In the group with diabetes, insulin-treated individuals had the highest risk of long-term mortality. In addition, in those with diabetes, higher quality of inpatient care was associated with lower risks of mortality; thus, this study highlights the importance of high-quality inpatient care in all individuals with diabetes.

## Supplementary Information

Below is the link to the electronic supplementary material.

ESM (PDF 320 KB)

## Data Availability

The data underlying this article were provided by the National Institute for Cardiovascular Outcomes Research (NICOR). Data will be shared on request to the corresponding author with the permission of NICOR.

## Abbreviations

ACS: Acute coronary syndrome
ACVC: Association for Acute Cardiovascular Care
AMI: Acute myocardial infarction
ARB: Angiotensin receptor blocker
CABG: Coronary artery bypass graft
CKD: Chronic kidney disease
COPD: Chronic obstructive pulmonary disease
ESC: European Society of Cardiology
ICA: Invasive coronary angiography
LMWH: Low molecular weight heparin
LVSD: Left ventricular systolic dysfunction
MACE: Major adverse cardiovascular events
MINAP: Myocardial Ischaemia National Audit Project
NSTEMI: Non-ST segment elevation myocardial infarction
OBQI: Opportunity-based quality indicator
ONS: Office for National Statistics
PCI: Percutaneous coronary intervention
