# Correction: Addressing disparities in the long-term mortality risk in individuals with non-ST segment myocardial infarction (NSTEMI) by diabetes mellitus status: a nationwide cohort study

**DOI:** 10.1007/s00125-024-06337-8

**Published:** 2024-12-20

**Authors:** Andrew Cole, Nicholas Weight, Shivani Misra, Julia Grapsa, Martin K. Rutter, Zbigniew Siudak, Saadiq Moledina, Evangelos Kontopantelis, Kamlesh Khunti, Mamas A. Mamas

**Affiliations:** 1 Keele Cardiovascular Research Group, Centre for Prognosis Research, Institute for Primary Care and Health Sciences, Keele University, Newcastle-under-Lyme, UK; 2 Division of Metabolism, Digestion and Reproduction, Faculty of Medicine, Imperial College London, London, UK; 3 Department of Diabetes, Endocrinology and Metabolism, Imperial College Healthcare NHS Trust, London, UK; 4 Cardiology Department, Guy’s and St Thomas’ NHS Foundation Trust, London, UK; 5 Diabetes, Endocrinology and Metabolism Centre, Manchester University NHS Foundation Trust, National Institute for Health and Care Research (NIHR) Manchester Biomedical Research Centre, Manchester, UK; 6 Division of Diabetes, Endocrinology and Gastroenterology, School of Medical Sciences, Faculty of Biology, Medicine and Health, University of Manchester, Manchester, UK; 7 Institute of Public Health, Jagiellonian University Medical College, Kraków, Poland; 8 Division of Informatics, Imaging and Data Sciences, University of Manchester, Manchester, UK; 9 Diabetes Research Centre, University of Leicester, Leicester, UK; 10 National Institute for Health and Care Research (NIHR) Birmingham Biomedical Research Centre, Birmingham, UK

**Correction: Diabetologia**

10.1007/s00125-024-06281-7

Shivani Misra’s name was spelt incorrectly in the author list. In addition, the affiliation for Zbigniew Siudak was incorrect.

The original article has been corrected.

