# Supplementary material for: Addressing disparities in the long-term mortality risk in individuals with non-ST segment myocardial infarction (NSTEMI) by diabetes mellitus status: a nationwide cohort study
Source: Diabetologia. 2024 Oct 3;67(12):2711–25. doi: 10.1007/s00125-024-06281-7 (PMC11604752; doi:10.1007/s00125-024-06281-7)
Supplement: Supplementary file 1 — ESM (PDF 320 KB) [file 125_2024_6281_MOESM1_ESM.pdf]

Addressing disparities in the long-term outcomes of non-ST segment myocardial infarction (NSTEMI) patients according to the presence of diabetes mellitus: a nationwide cohort study

Variable	No diagnosis of diabetes mellitus (n=346,121)	Diet-controlled diabetes (n=19,597)	Tablet-treated diabetes (n=58,828)	Insulin-dependent diabetes (n=34,130)	P-value
Age years	73.0 (61.2-82.6)	77 (67.5-84.1)	74.1 (64.6-81.1)	72.1 (63.2-79.3)	<0.001
Female	127,254/346,121 (37)	7,607/19,597 (39)	19,869/58,828 (34)	13,042/34,130 (38)	<0.001
BMI (kg/m^2)	26.6 (23.6-30.1)	27.7 (24.5-31.7)	28.4 (25.2-32.4)	29.6 (25.9-34.0)	<0.001
Ethnicity					
White	166,856/176,036 (95)	9,656/10,549 (92)	27,757/33,228 (84)	15,976/19,237 (83)	<0.001
Asian	7,312/176,036 (4)	755/10,549 (7)	4,758/33,228 (14)	2,689/19,237 (14)	
Black	1,521/176,036 (1)	120/10,549 (1)	608/33,228 (2)	419/19,237 (2)	
Mixed	347/176,036 (0)	18/10,549 (0)	105/33,228 (0)	54/19,237 (0)	
Killip Class					
Basal crepitations	22,283/159,997 (14)	1,859/9,700 (19)	5,490/30,990 (18)	3,796/17,648 (21)	<0.001
Pulmonary oedema	7,372/159,997 (5)	715/9,700 (7)	2,532/30,990 (8)	2,020/17,648 (12)	
Cardiogenic shock	824/159,997 (1)	70/9,700 (1)	170/30,990 (1)	135/17,648 (1)	
Grace Score					
High risk (>140)	94,326/155,824 (61)	6,849/9,443 (73)	20,591/30,142 (68)	12,201/17,164 (71)	<0.001
Intermediate risk (109–140)	41,031/155,824 (26)	1,914/9,443 (21)	6,914/30,142 (23)	3,712/17,164 (22)	
Low risk (<109)	20,467/155,824 (13)	680/9,443 (7)	2,637/30,142 (9)	1,251/17,164 (7)	
ECG ST changes	259,359/332,442 (78)	14,840/18,758 (79)	44,271/56,687 (78)	25,954/32,766 (79)	<0.001

Previous smoker	119,052/328,290 (36)	7,687/18,310 (42)	22,600/55,260 (41)	12,921/31,844 (41)	<0.001
Current smoker	77,583/328,290 (24)	3,090/18,310 (17)	9,709/55,260 (18)	4,823/31,844 (15)	
CCF	21,273/323,805 (7)	2,041/18,364 (11)	5,572/54,913 (10)	4,965/31,776 (15)	<0.001
Hypercholesterolemia	99,283/323,108 (31)	7,427/18,289 (42)	25,675/54,892 (47)	14,772/31,674 (47)	<0.001
Cerebrovascular disease	29,330/324,424 (9)	2,384/18,368 (13)	6,748/54,992 (12)	4,878/31,853 (15)	<0.001
Chronic renal failure	19,109/323,855 (6)	2,364/18,336 (13)	5,652/54,888 (10)	7,471/31,825 (23)	<0.001
History of angina	88,650/326,351 (27)	7,172/18,443 (38)	21,249/55,321 (38)	14,259/31,986 (44)	<0.001
Peripheral vascular disease	13,208/321,192 (4)	1,249/18,194 (7)	3,963/54,490 (7)	4,139/31,615 (13)	<0.001
Hypertension	163,361/329,020 (50)	12,396/18,691 (66)	39,216/55,967 (70)	22,570/32,351 (70)	<0.001
Asthma / COPD	55,067/322,265 (17)	3,616/18,299 (20)	10,066/54,750 (18)	6,253/31,735 (20)	<0.001
Previous acute myocardial infarction	78,021/329,477 (24)	6,324/18,574 (32)	19,106/55,819 (33)	14,153/32,271 (43)	<0.001
Previous PCI	32,095/324,775 (10)	2,470/18,280 (13)	8,652/54,987 (15)	6,502/31,762 (20)	<0.001
Previous CABG	21,100/325,304 (6)	1,900/18,366 (10)	6,611/55,185 (12)	5,219/31,917 (16)	<0.001
Family history of CAD	78,960/265,076 (30)	3,852/14,459 (26)	11,494/44,081 (26)	6,251/24,879 (25)	<0.001
Heart rate (bpm)	78 (66-92)	80 (68-94)	81 (70-96)	82 (70-97)	0.010
Systolic blood pressure (mmHg)	140 (121-159)	140 (122-160)	140 (122-159)	140 (122-160)	<0.001
LV function					
Good	92,970/244,381 (38)	4,574/13,752 (33)	15,121/43,060 (35)	8,032/24,750 (32)	<0.001
Moderate	40,064/244,381 (16)	2,522/13,752 (18)	8,712/43,060 (20)	5,522/24,750 (22)	
Severe	15,416/244,381 (6)	1,172/13,752 (9)	4,070/43,060 (9)	2,529/24,750 (10)	
Cardiac arrest	11,497/336,920 (3)	744/19,065 (4)	2,131/57,614 (4)	1,362/33,373 (4)	<0.001
Admission under cardiologist	150,829/337,998 (45)	8,312/19,137 (43)	25,712/57,370 (45)	14,744/33,268 (44)	0.011

Admission to cardiology ward	174,767/344,071 (51)	9,287/19,480 (47)	29,539/58,481 (50)	16,827/33,902 (51)	<0.001

ESM Table 1: Demographic comparison between patients with and without diabetes mellitus

CABG; coronary artery bypass graft, LVSD; left ventricular systolic dysfunction, CAD; coronary artery disease, COPD; chronic obstructive pulmonary disease, MI; myocardial infarction, CCF; congestive cardiac failure, BMI; body mass index, GRACE; global registry of acute coronary events. Admission to cardiology ward is a composite of admission to coronary care unit (CCU) and general cardiology ward. **Chronic kidney disease** is recorded in MINAP as those with serum creatinine chronically elevated above 200 micromol/L. Admission to cardiology ward refers to admission to either a coronary care unit or general cardiology ward. Continuous variables are expressed as median (IQR) and categorical variables as proportions (%). Denominators represent the total number of participants with a data point collected; numerators represent the number of those participants for whom the variable of interest was present

Variable	No diagnosis of diabetes mellitus (n=346,121)	Diet-controlled diabetes (n=18,689)	Tablet-treated diabetes (n=57,826)	Insulin-dependent diabetes (n=33,728)	P-value
LMWH	176,178/296,528 (59)	9,160/15,748 (58)	28,130/49,030 (57)	16,801/28,586 (59)	<0.001
Fondaparinux	106,038/256,384 (41)	5,811/13,823 (42)	18,499/43,808 (42)	9,814/25,420 (39)	<0.001
Warfarin	17,295/291,841 (6)	1,224/15,569 (8)	3,527/48,486 (7)	2,189/28,255 (8)	<0.001
Unfractionated heparin	39,297/290,561 (14)	1,981/15,527 (13)	6,689/48,245 (14)	4,211/28,135 (14)	<0.001
Glycoprotein 2b/3a inhibitor	12,621/296,924 (4)	540/15,775 (3)	1,736/49,223 (4)	941/28,682 (3)	<0.001
Intravenous nitrate	39,391/291,770 (14)	2,281/15,580 (15)	7,557/48,490 (16)	4,737/28,220 (17)	<0.001
Furosemide	77,111/292,893 (26)	5,972/15,660 (38)	18,990/48,778 (39)	14,344/28,494 (50)	<0.001
MRAs	12,941/215,599 (6)	971/11,623 (8)	3,385/37,903 (9)	2,479/21,943 (11)	<0.001
Aspirin	253,347/264,032 (96)	13,086/13,827 (94)	41,785/43,619 (96)	24,239/25,360 (96)	<0.001
$P2Y_{12}$ inhibitors	288,953/335,742 (86)	15,300/18,084 (85)	49,028/56,476 (87)	28,395/32,850 (86)	<0.001

Statins	279,390/339,291 (82)	15,574/18,318 (85)	50,881/56,827 (90)	29,732/33,108 (90)	<0.001
ACE inhibitors/ARBs	244,241/337,821 (72)	13,567/18,238 (74)	45,517/56,685 (80)	25,336/32,831 (77)	<0.001
β-blockers	240,625/336,559 (72)	13,030/18,193 (72)	42,142/56,535 (75)	24,241/32,847 (74)	<0.001
ICA	208,311/329,818 (63)	9,600/17,760 (54)	33,910/54,947 (62)	18,233/31,963 (57)	<0.001
PCI	101,933/275,233 (37)	4,407/14,503 (30)	15,998/45,806 (32)	8,306/26,039 (32)	<0.001
CABG surgery	9,216/275,233 (3)	557/14,503 (4)	2,327/45,806 (5)	1,118/26,039 (4)	<0.001
Revascularisation (CABG surgery/PCI)	111,149/275,233 (40)	4,964/14,503 (34)	18,325/45,806 (40)	9,424/26,039 (36)	<0.001
In-hospital mortality	20,177/346,132 (6)	1,519/18,689 (8)	3,789/57,826 (7)	2,421/33,728 (7)	<0.001
30 day mortality	24,034/346,132 (7)	1,778/18,689 (10)	4,625/57,826 (8)	3,000/33,728 (9)	0.008
1 year mortality	60,572/346,132 (18)	4,717/18,689 (25)	12,202/57,826 (21)	8,939/33,728 (27)	<0.001
5 year mortality	104,670/276,101 (38)	7,746/15,207 (51)	21,384/44,661 (48)	15,302/26,042 (48)	<0.001
10 year mortality	84,286/151,055 (56)	5,744/8,101 (71)	15,659/22,111 (71)	10,375/13,039 (80)	<0.001
Inpatient cardiac mortality	15,355/346,132 (4)	1,153/18,689 (6)	2,996/57,826 (5)	1,882/33,728 (6)	0.001
Reinfarction	3,178/312,290 (1)	210/17,081 (1)	615/52,606 (1)	417/30,533 (1)	<0.001
Major bleeding	5,102/334,717 (1)	316/18,250 (2)	978/56,278 (2)	580/32,751 (2)	<0.001
MACE[a]	176,178/296,528 (59)	1,670/18,689 (9)	4,253/57,826 (7)	2,720/33,728 (8)	<0.001
Circulatory cause of death	106,038/256,384 (41)	5,813/11,567 (50)	17,292/33,382 (52)	12,719/23,105 (55)	<0.001

ESM Table 2: Management strategy and clinical outcome comparison between NSTEMI patients with and without diabetes mellitus
IV; intravenous, MRA; mineralocorticoid receptor antagonist, ACE: angiotensin-converting-enzyme, ARB; angiotensin receptor blockers, CABG; coronary artery bypass graft, PCI; percutaneous coronary intervention and MACE; major adverse cardiovascular events. MACE is defined as the composite endpoint of in-hospital death and reinfarction. **Chronic kidney disease** is recorded in MINAP as those with serum

creatinine chronically elevated above 200 micromol/L. “Long-term cardiovascular mortality” variable refers to death from a cardiovascular cause as defined by the Office of National Statistics (ONS) in entire duration of the study. Continuous variables are expressed as median (IQR) and categorical variables as proportions (%). Denominators represent the total number of participants with a data point collected; numerators represent the number of those participants for whom the variable of interest was present

Variables	No diagnosis of diabetes mellitus (n=346,121)	Diet-controlled diabetes (n=18,689)	Tablet-treated diabetes (n=57,826)	Insulin-dependent diabetes (n=33,728)	P-value
Coronary Angiography received within 72 hours	35,278/53,243 (66)	1,381/2,367 (58)	5,117/8,667 (59)	2,479/4,675 (53)	<0.001
LV Function recorded in notes	193,444/316,884 (61)	7,861/13,066 (60)	27,467/42,310 (65)	15,828/24,380 (64)	<0.001
Fondaparinux or LMWH received	330,666/379,356 (87)	13,318/15,441 (86)	41,469/48,390 (86)	23,594/28,100 (84)	<0.001
DAPT received on discharge	220,566/274,613 (80)	11,163/14,444 (77)	36,848/45,599 (81)	21,246/26,500 (80)	<0.001
ACEi or ARB on discharge for those with moderate and severe LVSD	53,829/69,989 (77)	2,649/3,446 (77)	10,273/12,422 (83)	6,190/7,808 (79)	<0.001
Beta Blocker on discharge for those for those with moderate and severe LVSD	52,778/70,025 (75)	2,532/3,364 (75)	9,194/11,949 (77)	5,852/7,519 (78)	<0.001
Composite All/None score**	289,175/424,640 (68)	10,449/15,676 (67)	35,201/48,327 (73)	20,119/27,894 (72)	<0.001
Composite All/None score for those with moderate and severe LVSD***	34,554/49,181 (70)	2,094/2,962 (71)	7,892/10,518 (75)	4,947/6,616 (75)	<0.001
Mean OBQI score	79.3	79.2	82.4	81.4	<0.001
Cardiac rehabilitation	323,303/405,761 (80)	12,430/16,920 (74)	40,105/52,118 (77)	22,748/30,436 (75)	<0.001

ESM Table 3: Quality Indicators for NSTEMI patients with and without diabetes mellitus (ESC ACVC and OBQI)
Denominators represent total number of patients with a datapoint collected, numerators represent the numbers of these where the variable of interest was present.

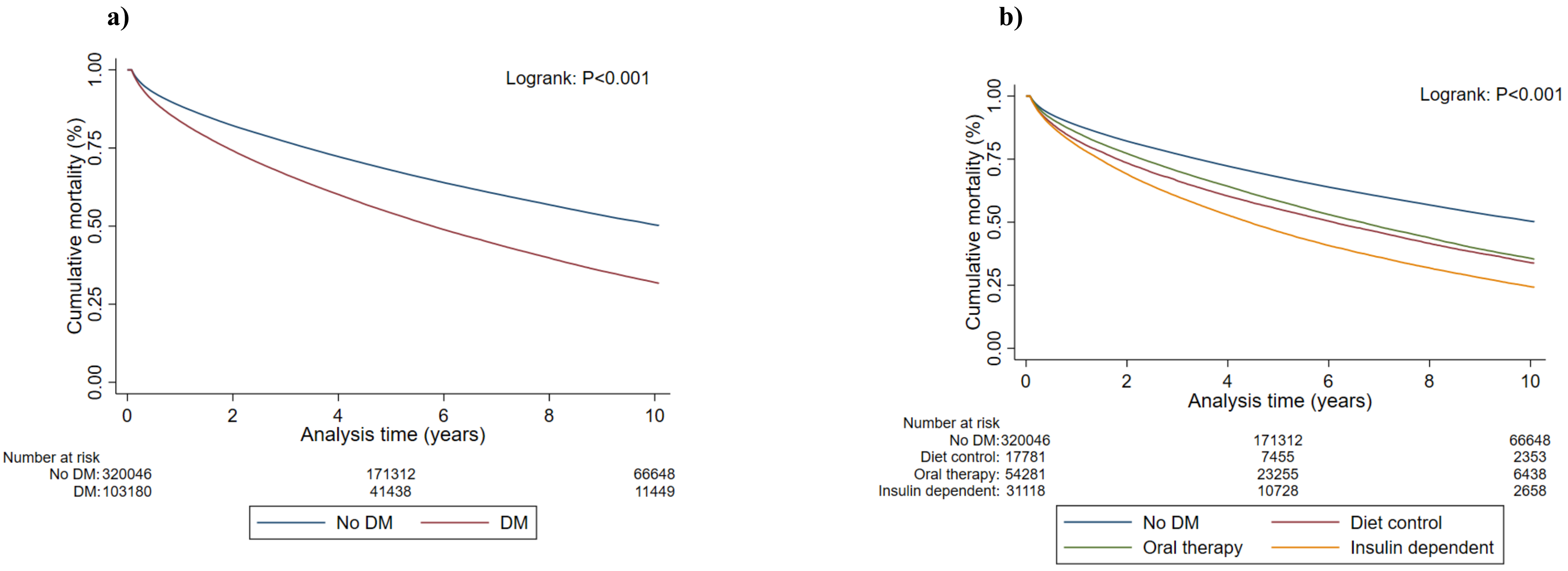

ESM Figure 1: Landmark Kaplan-Meier Survival Curves for Diabetes mellitus patients compared to those without (mortality within 30d of index admission with NSTEMI excluded)

a) All DM patients
b) Subdivided groups based on used of glycaemic control measures
